# Predictors of hospital surface contamination with Extended-spectrum β-lactamase-producing *Escherichia coli* and *Klebsiella pneumoniae*: patient and organism factors

**DOI:** 10.1186/2047-2994-3-5

**Published:** 2014-02-04

**Authors:** Joshua T Freeman, Jessica Nimmo, Eva Gregory, Audrey Tiong, Mary De Almeida, Gary N McAuliffe, Sally A Roberts

**Affiliations:** 1 Department of Clinical Microbiology, Auckland District Health Board, Auckland, New Zealand; 2 Faculty of Medical and Health Sciences, University of Auckland, 22 Princes Street, Auckland 1010, New Zealand

## Abstract

**Background:**

The role of the hospital environment in transmission of ESBL-*Klebsiella pneumoniae* (ESBL-KP) and ESBL-*Escherichia coli* (ESBL-EC) is poorly defined. Recent data however suggest that in the hospital setting, ESBL-KP is more transmissible than ESBL-EC. We sought therefore to measure the difference in hospital contamination rates between the two species and to identify key risk factors for contamination of the hospital environment with these organisms.

**Methods:**

We systematically sampled 8 surfaces in the rooms and bathrooms of adult patients colonized or infected with ESBL-EC or ESBL-KP throughout their hospital stay. Data were collected on factors potentially affecting contamination rates. Environmental contamination was defined as recovery of an ESBL-producing organism matching the source patient’s isolate. Multivariate logistic regression analysis was performed at the level of the patient visit using generalized estimating equations to identify independent predictors of environmental contamination.

**Results:**

24 patients (11 with ESBL-KP, 11 ESBL-EC and 2 with both organisms) had 1104 swabs collected during 138 visits. The overall contamination rate was 3.4% (38/1104) and was significantly higher for ESBL-KP than ESBL-EC (5.4% versus 0.4%; p < 0.0001). After multivariate analysis, environmental contamination was found to be negatively associated with carbapenem exposure (OR 0.06 [95% CI 0.01-0.61]; p = 0.017) and positively associated with the presence of an indwelling urinary catheter (OR 6.12 [95% CI 1.23-30.37]; p = 0.027) and ESBL-KP in the source patient (OR 26.23 [95% CI 2.70-254.67]; p = 0.005).

**Conclusions:**

Contamination of the hospital environment with ESBL-producing Enterobacteriaceae (ESBL-E) is inversely associated with carbapenem exposure. Predictors of hospital contamination with ESBL-E include: indwelling urinary catheters and ESBL-KP. Rooms of patients with ESBL-KP have substantially higher contamination rates than those with ESBL-EC. This finding may help explain the apparently higher transmissibility of ESBL-KP in the hospital setting.

## Background

The prevalence of extended spectrum β-lactamase producing Enterobacteriaceae (ESBL-E) has increased sharply over the last decade [1,2]. The reasons for this are complex, but transmission in healthcare settings is thought to be an important contributing factor [3,4]. To reduce transmission risk, many hospitals assign colonized or infected patients to a single room and use gowns and gloves for all patient contact [5]. This approach is based on the assumption that contamination of clothing and the hospital environment play an important role in the transmission of ESBL-E species [6]. For many healthcare-associated pathogens the degree of hospital surface contamination is closely correlated with the risk of transmission*,* but the extent to which this is true for ESBL-EC and ESBL-KP remains uncertain [7-9]. Recent studies however do suggest that rates of transmission in the hospital setting are higher for ESBL- *K. pneumoniae* (ESBL-KP) than ESBL-*E. coli* (ESBL-EC) [10-13]. In light of these observations, we performed a prospective cohort study to determine whether rates of contamination of the hospital environment are correspondingly higher for ESBL-KP than ESBL-EC. Secondly, we recorded a variety of patient factors that could potentially affect contamination rates, in order to identify key patient and organism factors that affect the risk of hospital surfaces becoming contaminated with ESBL-E.

## Methods

### Design, setting and recruitment

Recruitment of patients took place between 13th November 2012 and 15th January 2013 at Auckland City Hospital (ACH). ACH is a tertiary level institution with 700 beds for adult patients. For inclusion in the study, adult patients admitted to ACH were required to have ESBL-EC or ESBL-KP recovered from either rectal swabs or clinical specimens during their hospital stay. Eligible patients were also required to give informed consent and to be sufficiently mobile to use the bathroom facilities. According to hospital policy, rectal swabs are collected on all patients with a history of hospital admission during the preceding year in order to screen for ESBL-E colonization. Patients found to be colonized with either ESBL-EC or ESBL-KP are then managed with contact precautions in a single room. Patients admitted to the maternity and psychiatric wards as well as intensive care units were excluded. Those meeting criteria for inclusion were asked to give informed consent to participate in the study. Participation involved collection of serial sets of environmental swabs as well as data on putative risk factors for environmental contamination from the patient records. Environmental sampling was performed daily on week days in the morning. Standard hospital cleaning protocols used throughout the study period consisted of once daily cleaning of the over bed tables, call bells and bedside cabinets with a damp cloth and detergent plus twice daily cleaning of toilets, basins and high touch surfaces in the patient bathrooms using 0.1% sodium hypochlorite solution. Over weekends there was a 3 day interval between sampling. Cleaning staff were not informed about the study. Sampling continued until patient discharge. For each patient visit, whether or not cleaning had occurred previously on the same day was recorded. Ethics approval for this study was granted by the Northern Regional Ethics Committee.

### Data collection

For each patient admission the following putative risk factors for environmental contamination were collected from the clinical records: age and gender of patient; the use of an indwelling urinary catheter (IDC); the receipt of antibiotics in general as well as the receipt of carbapenem antibiotics specifically; the Chronic Disease Score–Infectious Diseases (CDS-ID) score [14]; whether the patient had at least one documented episode of diarrhoea; the specimen site (s) from which ESBL-E were recovered during their stay; the clinical service caring for the patient and whether or not the patient had clinical infection with ESBL-E. At each visit, whether or not the room had undergone cleaning that day prior to environmental sampling was also recorded. The primary outcome of interest was environmental contamination with ESBL-E. Environmental contamination was defined as recovery of an environmental ESBL-E with the same species and antibiotic susceptibility profile as the patient’s isolate.

### Environmental sampling

Sampling of the environment was carried out in eight areas in the patient room and bathroom. An approximate 10 cm by 10 cm square was sampled with five longitudinal and five latitudinal strokes of a sterile, nylon tipped flocked swab (Copan diagnostics, California, USA) pre-moistened with nutrient broth immediately before use. This was then placed in 1.5 mL of nutrient broth in sterile containers, and swirled for 20 seconds. Samples were then vortexed for 2 minutes before being incubated overnight at 35°C. The eight environmental surfaces sampled were as follows: the blood pressure cuff; the nurses’ call bell; the top of the patient’s bedside cabinet; the patient’s over bed tray table; the toilet seat; the hand rail next to the toilet; the basin tap in the bathroom and the bathroom’s inside door handle. In addition to the eight environmental samples, the patient’s antecubital fossa was also sampled at each visit.

### Culture methods

After being incubated overnight the nutrient broth samples were recorded to show either growth or no growth, based on the presence of a turbid solution. The samples showing growth were then plated on MacConkey (MAC) agar and incubated overnight at 35°C. Any colonies on MAC agar were subcultured to ChromID® ESBL agar (bioMerieux, Marcy L’Etoile, France) and incubated overnight at 35°C. Colonies on chromogenic agar were identified according to routine laboratory protocols using Matrix Assisted Laser Desorption/Ionization-Time of Flight, (MALDI-TOF), mass spectrometry (Vitek MS, bioMerieux). ESBL status was confirmed using the CLSI combined disk diffusion test. Susceptibility testing was performed using the Vitek II® (bioMerieux) gram negative susceptibility card AST-N247 against a range of 12 antibiotics (meropenem, trimethoprim-sulphamethoxazole, amoxicillin-clavulanate, ciprofloxacin, norfloxacin, cefoxitin, gentamicin, amikacin, nitrofurantoin, piperacillin-tazobactam, tobramycin).

### Statistical analysis

Analysis was performed using each patient visit as a data point. The dependent variable was recovery from at least one environmental site of an ESBL-producing organism with the same species and susceptibility profile as the patient’s isolate. Fisher’s exact and the unpaired Wilcoxon rank sum test were used to compare categorical and continuous variables respectively. For each patient, visits were numbered sequentially. “Visit number” was included in the analysis as a continuous variable to determine whether the probability of detecting contamination changed with successive visits. Significant patient and organism factors on univariate analysis were included in a multivariate model derived using backward stepwise logistic regression. The multivariate model was derived using generalized estimating equations to control and adjust for the possibility that outcomes associated with repeated visits to individual patients may not have been independent. The significance of individual predictors during fitting of the model was assessed using the Wald statistic. All testing was two tailed and statistical significance was defined as P ≤ 0.05. Statistical analysis was performed using SPSS software (Version 21.0).

## Results

### Patient characteristics

Between 13th November 2012 and 15th January 2013, 24 of 46 (52%) eligible patients gave informed consent to participate in the study. The remaining 22 patients either declined to participate, or were discharged prior to obtaining consent, or were unable to give informed consent due to impaired cognition or language barriers. For the 24 participating patients (11 with ESBL-EC, 11 with ESBL-KP and 2 patients co-colonized with both organisms), 1104 environmental swabs were collected during a total of 138 visits. Of the 24 participants, 17 were recruited on the basis of positive rectal swabs (10 with ESBL-EC, 5 with ESBL-KP and two with both species) and 7 were recruited on the basis of a positive clinical specimen (6 with ESBL-KP and 1 with ESBL-EC). Positive clinical specimens included catheter urine specimens from 2 patients; a mid stream urine from one patient; wound swabs from 3 patients and one patient with a positive blood culture collected from a central line. These corresponded to five clinical infections: two catheter-associated UTIs (one ESBL-EC, one ESBL-KP); one urinary infection without an IDC (ESBL-KP); one surgical wound infection (ESBL-KP) and one central line infection (ESBL-KP). Of the 138 visits; 52 were to patients with ESBL-EC, 81 were to patients with ESBL-KP and 5 were to patients colonized with both organisms. Patients were under the care of a variety of surgical and medical services: cardiothoracic and vascular surgery- 4 patients; general medicine and elderly care- 6; haematology and oncology- 4; respiratory-2; cardiology-2; and one patient each from neurology, neurosurgery, head and neck surgery, renal, urology and orthopaedics. No significant associations were observed between environmental contamination and any particular clinical service (data not shown).

### Rates and distribution of contamination

**Figure 1:**
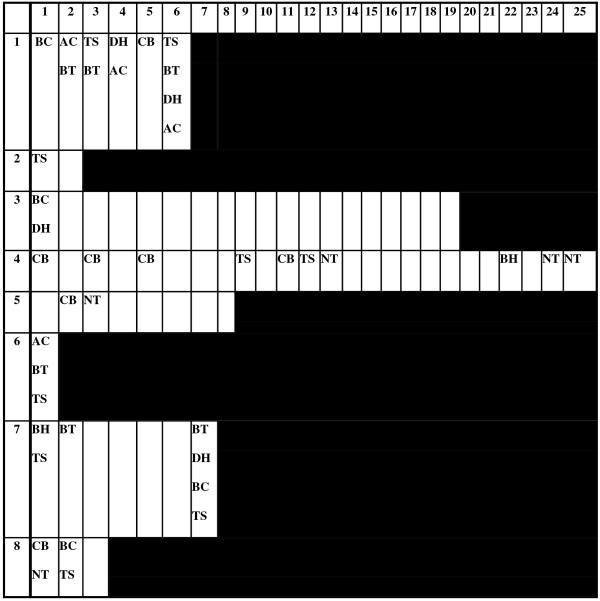
**Time line of visits for the 8 patients with at least one positive environmental swab.** Figure 1 Legend: Rows 1–8 represent patients with at least one positive environmental sample. Columns 1–25 indicate visit number. Blank cells represent visits for which no environmental contamination was detected. BT = Basin tap, TS = Toilet seat, DH = Bathroom door handle, AC = Antecubital fossa, CB = Call bell, NT = Nightingale (over bed) table, BC = Bedside cabinet, BH = bathroom handrail. *Patient 2 was colonized with ESBL-EC alone and patient 8 was colonized with both ESBL-EC and ESBL-KP. The remaining patients were colonized with ESBL-KP alone.

Overall, the rate of environmental contamination with ESBL-E was 38/1104 (3.4%). One environmental swab yielded both organisms from a patient co-colonized with ESBL-EC and ESBL-KP. There were no cases where the environmental isolate (s) did not match the species of the corresponding isolate (s) from the room occupant. Corresponding isolates also had consistent antibiotic susceptibility profiles against all 12 antibiotics tested. The 38 environmental isolates were obtained from 8 patients during 26/138 visits. Thus, there were a total of 112 visits where no environmental contamination was detected. There was no obvious relationship between sites of contamination on consecutive visits for individual patients (Figure 1). The interval between consecutive visits was either 1 day during the week or 3 days over weekends except for an extended interval (during the New Year period) of 17 days for patient 4 between visits 19 and 20 (Figure 1). When examined by environmental site, the rate of environmental recovery was significantly higher for ESBL-KP than for ESBL-EC (37/688 [5.4%] versus 2/456 [0.4%] respectively; p < 0.0001 [co-colonized patients included in both groups]). Similar findings were seen when analysed at the visit level (Table 1).

**Table 1:** Univariate analysis comparing 26 visits with environmental contamination with the 112 visits without environmental contamination

	**Contaminated (%)**	**Non- contaminated (%)**	**Crude OR (95%CI)**	**P value**
Room cleaned	3/26 (11.5)	20/112 (17.9)	0.60 (0.13–2.40)	0.57
Median visit number (IQR)	3 (1–9)	4 (2–8)	–	0.59
ESBL-KP	25/26 (96.2)	61/112 (54.5)	20.90 (2.85–428.64)	<0.0001
CDS-ID ≥ 1	22/26 (84.6)	86/112 (76.8)	1.66 (0.48–6.29)	0.44
Median age (IQR)	75 (62–86)	66 (48–81)	–	0.05
Male patient	22/26 (84.6)	51/112 (45.5)	6.58 (1.97–24.24	<0.0001
Patient 4	10/26 (38.5)	15/112 (13.4)	4.04 (1.40–11.72)	0.008
Patient 1	6/26 (23.1)	0/112 (0)	–	<0.0001
Antibiotic exposure	11/26 (42.3)	79/112 (70.5)	0.31 (0.12–0.80)	0.01
Carbapenem exposure	3/26 (11.5)	40/112 (35.7)	0.24 (0.05–0.90)	0.02
IDC	14/26 (53.8)	26/112 (23.2)	3.86 (1.46–10.29)	0.003
Diarrhoea	8/26 (30.8)	12/112 (10.7)	3.70 (1.18–11.58)	0.025
Clinical specimen ESBL-E	14/26 (53.8)	51/112 (45.5)	1.40 (0.55–3.56)	0.52
Clinical infection	3/26 (11.5)	34/112 (30.4)	0.30 (0.07–1.15)	0.05
Isolation from wound swab	12/26 (46.2)	33/112 (29.5)	2.05 (0.79–5.34)	0.11
Isolation from urine	2/26 (7.7)	11/112 (9.8)	0.77 (0.11–4.05)	1.0

“IQR” = interquartile range; “Clinical Specimen ESBL-E” = at least one positive clinical specimen culture positive with ESBL-E.

**Figure 2:**
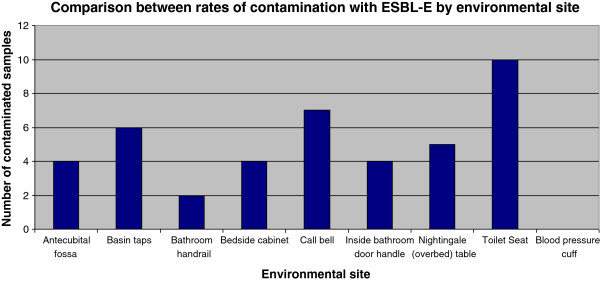
Relative rates of contamination across the different sites sampled.

The number of samples positive for ESBL at each of the eight environmental sites plus the antecubital fossa is shown in Figure 2. Two patients colonized with ESBL-KP alone had ESBL-KP isolated from the antecubital fossa during 3 visits for one patient and 1 visit for the other [4/138 (2.9%)].

### Risk factor analysis

Factors associated with environmental contamination on univariate analysis are shown in Table 1. Along with a variety of other factors, the “patient identity” of two patients was associated with environmental contamination (patients 1 and 4, Figure 1). Patient 1 was a 75 year old male under the care of the vascular surgery service who had an IDC in place throughout his stay and had ESBL-KP isolated from a rectal swab only. Patient 4 was an 86 year old male under the care of the head and neck surgery service with no IDC and ESBL-KP isolated from a surgical scalp wound but was not thought to be causing clinical infection. A negative association was observed with antibiotic use in general as well as carbapenem use specifically. A negative association between environmental contamination and clinical infection due to ESBL-E was also observed. Three of the five patients with clinical infection received treatment with a carbapenem.

No significant difference between contamination rates were observed according to whether the patient was recruited on the basis of a positive clinical specimen; or more specifically, whether or not the organism was isolated from urine or a wound swab. There was no significant change in the likelihood of contamination with successive visits. Because of the high level of co-linearity between clinical infection and carbapenem exposure, only the latter variable was included in the multivariate model based on its lower p value on univariate analysis. Variables that remained significantly associated with environmental contamination in the final multivariate model were: colonization with ESBL-KP (Odds ratio [OR] 26.23 [95% confidence interval (CI): 2.70-254.67]; p = 0.005); the use of an IDC in the source patient (OR 6.12 [95% CI: 1.23-30.37]; p = 0.027) and carbapenem exposure (OR 0.06 [95% CI 0.01-0.61]; p = 0.017) (Table 2). There was also a positive association with male gender although this was not quite statistically significant (OR 10.53 [95% CI 0.83-133.56]; p = 0.069).

**Table 2:** Factors remaining significantly associated with environmental contamination in the multivariate model

	**Adjusted OR (95%CI)**	**P value**
ESBL-KP	26.23 (2.70–254.67)	0.005
Indwelling urinary catheter	6.12 (1.23–30.37)	0.027
Carbapenem exposure	0.06 (0.01–0.61)	0.017

ESBL-KP = ESBL-*Klebsiella pneumoniae* (Referenced to ESBL-*Escherichia coli*).

## Discussion

Our findings suggest that patients with ESBL-E are more likely to contaminate their hospital room with viable ESBL-E if they are colonized or infected with ESBL-KP rather than ESBL-EC. Moreover, our findings suggest that this difference between species cannot be accounted for by corresponding differences between patient characteristics alone. Even after we adjusted for a wide range of potential confounding factors by multivariate analysis, the association between ESBL-KP and environmental contamination remained highly significant. One possible explanation for this finding is that intrinsic biological differences between *E. coli* and *K. pneumoniae* affect their capacity to remain viable in the environment; a conclusion for which there is at least some supportive data [15,16]. Another possibility is that the particular cleaning practices at our hospital had a lesser impact on ESBL-KP than ESBL-EC although this possibility is undermined by the similarity between our findings and those of a recent study performed in a pediatric hospital in France [17]. In that study also, ESBL-KP remained a significant risk factor for environmental contamination even after adjusting for host factors by multivariate analysis. Similar findings have also been reported by investigators from another French hospital [18]. It appears therefore that differences in hospital contamination rates between the two species are consistent between different geographic regions, between different patient groups and between different hospitals with different cleaning practices. When taken together therefore, these findings may potentially help to explain the growing body of evidence indicating that in the hospital setting, ESBL-KP has greater transmission potential than ESBL-EC [10-13].

Our study also identified risk factors associated with the source patient. Firstly, we found the association between environmental contamination and the source patient having an indwelling urinary catheter (IDC) remained significant after multivariate analysis. This finding suggests that urinary catheters can play an important role in facilitating environmental contamination and that this association is independent of the patient having a catheter associated urinary tract infection. More work is needed to corroborate this finding but on a practical level, this finding reinforces the importance of programs to reduce unnecessary IDC use and to ensure their removal at the earliest opportunity [19].

We also observed an association between male gender and environmental contamination that trended towards significance in the final multivariate model; possibly reflecting gender-based differences in hygiene practices. Of note, male gender has been reported to be an independent risk factor for both ESBL-infection and colonization in several previous studies [20-22]. We also found that patients exposed to antibiotics (and more specifically carbapenems) had lower rates of contamination. This suggests that some antibiotics such as carbapenems with activity against ESBL-E may reduce bacterial load of the organism as has been reported previously for VRE [23]. It seems very likely that the negative association we observed between environmental contamination and clinical infection is also explained by the same mechanism.

In keeping with the French pediatric study, prior cleaning did not appear to have a significant effect on contamination rates, perhaps because cleaning was inadequate and/or there was a tendency for contamination to reoccur rapidly between cleans. High rates of contamination were found on call bells and over bed tables in keeping with reports that some of the most heavily contaminated surfaces are those closest to the patient bed [24,25].

Our study has both strengths and weaknesses that deserve discussion. Firstly, although we were able to directly compare rates of contamination between ESBL-EC and ESBL-KP, we could not assess the risk of transmission posed by the levels of contamination we detected. Secondly the number of visits for each patient was not uniform. However on multivariate analysis, we found no significant relationship between contamination and any particular patient, indicating that these factors were less predictive than others we investigated. Thirdly, we did not collect data on possible differences in adherence to standard cleaning protocols between wards. Finally, we did not perform molecular typing to conclusively establish that the environmental isolates and plasmids were identical to those from the patient. However, the corresponding species and extensive antimicrobial susceptibility profiles were consistent between corresponding patient and environmental isolates without exception. The strengths of the study include the large number of environmental samples; the longitudinal sampling (allowing assessment of change in risk of contamination over time); the assessment of the relative importance of both patient and organism risk factors; the range of relevant risk factors that were investigated and the current lack of published data on factors predisposing to environmental contamination with ESBL-E.

## Conclusions

Hospital environmental contamination rates are substantially higher for patients with ESBL-KP compared to those with ESBL-EC. This observation may help explain corresponding differences in transmission rates between the two organisms. Patients receiving carbapenems have reduced contamination rates whereas indwelling urinary catheters increase the likelihood of the hospital environment becoming contaminated with ESBL-E. Further studies are needed to confirm the external validity of our findings. Improved understanding of the important mechanisms by which ESBL-E transmit between patients in the hospital setting will provide an opportunity to develop new strategies to prevent their transmission.

## Competing interests

The authors declare that they have no competing interests.

## Authors’ contributions

JTF contributed to the concept and design of the study, the statistical analysis and manuscript preparation. JN and EG were involved in obtaining patient consent, collecting specimens and collecting patient data. AT, MD and GNM were involved in processing microbiological samples, susceptibility testing and species identification as well as manuscript review. SAR contributed to the concept and design, organization and execution of the study, as well as review of the manuscript. All authors read and approved the final manuscript.

## References

[B1] Hoban DJ Bouchillon SK Hawser SP Badal RE Trends in the frequency of multiple drug-resistant Enterobacteriaceae and their susceptibility to ertapenem, imipenem and other antimicrobial agents: data from the Study for Monitoring Antimicrobial Resistance Trends 2002–2007 Diagn Microbiol Infect Dis 2010 3 78 86 10.1016/j.diagmicrobio.2009.06.009 19733993

[B2] Hawser SP Badal RE Bouchillon SK Monitoring the global in vitro activity of ertapenem against *Escherichia coli* from intra-abdominal infections: SMART 2002–2010 Int J Antimicrob Agents 2013 3 224 228 10.1016/j.ijantimicag.2012.10.014 23305657

[B3] Oteo J Navarro C Cercenado E Spread of *Escherichia coli* strains with high-level cefotaxime and ceftazidime resistance between the community, long term care facilities and hospital institutions J Clin Microbiol 2006 3 2359 2366 10.1128/JCM.00447-06 16825350 PMC1489527

[B4] Rodriguez-Bano J Picon E Gijo P Community-onset bacteraemia due to extended-spectrum beta-lactamase-producing *Escherichia coli*: risk factors and prognosis Clin Infect Dis 2010 3 40 48 10.1086/649537 19995215

[B5] Siegel JD Rhinehart E Jackson M Chiarello L Management of multidrug-resistant organisms in healthcare settings, 2006 Am J Infect Control 2007 3 165 193 10.1016/j.ajic.2007.10.006 18068814

[B6] Freeman JT Williamson DA Anderson DJ When should contact precautions and active surveillance be used to manage patients with multidrug-resistant Enterobacteriaceae? Infect Control Hosp Epidemiol 2012 3 753 756 10.1086/666333 22669239

[B7] Nseir S Blazejewski C Lubret R Wallet F Courcol R Durocher A Risk of acquiring multidrug-resistant gram-negative bacilli from prior room occupants in the intensive care unit Clin Microbiol Infect 2011 3 1201 1208 10.1111/j.1469-0691.2010.03420.x 21054665

[B8] Weber DJ Rutala WA Understanding and preventing transmission of healthcare-associated pathogens due to the contaminated hospital environment Infect Control Hosp Epidemiol 2013 3 449 452 10.1086/670223 23571359

[B9] Adebola OA Johnson JK Harris AD Risk of acquiring extended-spectrum β-lactamase-producing *Klebsiella* species and *Escherichia coli* from prior room occupants in the intensive care unit Infect Control Hosp Epidemiol 2013 3 453 458 10.1086/670216 23571360 PMC3660030

[B10] Hilty M Betsch BY Bogli-Stuber K Transmission dynamics of extended-spectrum β-lactamase-producing Enterobacteriaceae in the tertiary care hospital and the household setting Clin Infect Dis 2012 3 967 975 10.1093/cid/cis581 22718774 PMC3436924

[B11] Harris AD Kotetishvili M Shurland S How important is patient to patient transmission in extended-spectrum β-lactamase *Escherichia coli* acquisition Am J Infect Control 2007 3 97 101 10.1016/j.ajic.2006.09.011 17327188

[B12] Harris AD Perencevich EN Johnson JK Patient to patient transmission is important in extended-spectrum β-lactamase producing *Klebsiella pneumoniae* acquisition Clin Infect Dis 2007 3 1347 1350 10.1086/522657 17968833

[B13] Cholley P Thouverez M Gbaguidi-Haore H Hospital cross-transmission of extended-spectrum β-lactamase *Escherichia coli* and *Klebsiella pneumoniae* Med Mal Infect 2013 3 331 336 10.1016/j.medmal.2013.06.001 23876202

[B14] McGregor JC Kim PW Perencevich EN Utility of the chronic disease score and charlson comorbidity index as comorbidity measures for use in epidemiologic studies of antibiotic-resistant organisms Am J Epidemiol 2005 3 483 493 10.1093/aje/kwi068 15718484

[B15] Kramer A Schwebke I Kampf G How long do nosocomial pathogens persist on inanimate surfaces? A systematic review BMC Infect Dis 2006 3 130 10.1186/1471-2334-6-130 16914034 PMC1564025

[B16] Neely AN A survey of gram negative bacteria survival on hospital fabrics and plastics J Burn Care Rehabil 2000 3 523 527 10.1097/00004630-200021060-00009 11194806

[B17] Guet-Rivellet H Le Monnier A Bretton N Environmental contamination with extended-spectrum β-lactamases: is there any difference between *Escherichia coli* and *klebsiella* spp? Am J Infect Control 2012 3 845 848 10.1016/j.ajic.2011.10.007 22325483

[B18] Gbaguidi-Haore H Talon D Hocquet D Bertrand X Hospital environmental contamination with Enterobacteriaceae producing extended-spectrum β-lactamase Am J Infect Control 2013 3 664 665 23337304 10.1016/j.ajic.2012.07.021

[B19] Meddings J Rogers MA Macy M Saint S Systematic review and meta-analysis: reminder systems to reduce catheter-associated urinary tract infections and urinary catheter use in hospitalized patients Clin Infect Dis 2010 3 550 560 10.1086/655133 20673003

[B20] Ben Ami R Schwaber MJ Nanon-Venezia S Influx of extended-spectrum β-lactamase-producing Enterobacteriaceae into the hospital Clin Infect Dis 2006 3 925 934 10.1086/500936 16511754

[B21] Hayakawa K Gattu S Marchaim D Epidemiology and risk factors for isolation of *Escherichia coli* producing CTX-M-type extended-spectrum β-lactamase in a large US medical center Antimicrob Agents Chemother 2013 3 4010 4018 10.1128/AAC.02516-12 23752516 PMC3719715

[B22] Kang CI Wi YM Lee MY Epidemiology and risk factors of community onset infections caused by extended-spectrum β-lactamase-producing *Escherichia coli* strains J Clin Microbiol 2012 3 312 317 10.1128/JCM.06002-11 22162561 PMC3264158

[B23] Donskey CJ Chowdhry TK Hecker MT Effect of antibiotic therapy on the density of vancomycin resistant enterococci in the stool of colonized patients N Engl J Med 2000 3 1925 1932 10.1056/NEJM200012283432604 11136263 PMC4370337

[B24] Moore G Muzslav M Wilson PR The type, level and distribution of microorganisms within the ward environment: a zonal analysis of an intensive care unit and gastrointestinal surgical ward Infect Control Hosp Epidemiol 2013 3 500 506 10.1086/670219 23571367

[B25] Judge C Galvin S Burke L Thomas T Humphreys H Fitzgerald-Hughes D Search and you will find: detecting extended-spectrum β-lactamase producing *Klebsiella pneumoniae* from a patient’s immediate environment Infect Control Hosp Epidemiol 2013 3 534 536 10.1086/670206 23571375

